# Association of asthma genetic variants with asthma‐associated traits reveals molecular pathways of eosinophilic asthma

**DOI:** 10.1002/clt2.12239

**Published:** 2023-04-20

**Authors:** Zaid W. El‐Husseini, Judith M. Vonk, Maarten van den Berge, Reinoud Gosens, Gerard H. Koppelman

**Affiliations:** ^1^ Department of Pediatric Pulmonology and Pediatric Allergology Beatrix Children's Hospital University of Groningen University Medical Center Groningen Groningen The Netherlands; ^2^ Groningen Research Institute for Asthma and COPD (GRIAC) University of Groningen University Medical Center Groningen Groningen The Netherlands; ^3^ Molecular Pharmacology Groningen Research Institute of Pharmacy Groningen The Netherlands; ^4^ Department of Epidemiology University of Groningen University Medical Center Groningen Groningen The Netherlands; ^5^ Department of Pulmonary Diseases University of Groningen University Medical Center Groningen Groningen The Netherlands

**Keywords:** asthma heterogeneity, endotype, genome wide association, phenotype, single nucleotide polymorphism

## Abstract

**Introduction:**

Asthma is a complex, polygenic, heterogenous inflammatory disease. Recently, we generated a list of 128 independent single nucleotide polymorphisms (SNPs) associated with asthma in genome‐wide association studies. However, it is unknown if asthma SNPs are associated with specific asthma‐associated traits such as high eosinophil counts, atopy, and airway obstruction, revealing molecular endotypes of this disease. Here, we aim to identify the association between asthma SNPs and asthma‐associated traits and assess expression quantitative trait locus (e‐QTLs) to reveal their downstream functional effects and find drug targets.

**Methods:**

Association analyses between 128 asthma SNPs and associated traits (blood eosinophil numbers, atopy, airway obstruction, airway hyperresponsiveness) were conducted using regression modelling in population‐based studies (Lifelines *N* = 32,817/Vlagtwedde‐Vlaardingen *N* = 1554) and an asthma cohort (Dutch Asthma genome‐wide association study *N* = 917). Functional enrichment and pathway analysis were performed with genes linked to the significant SNPs by e‐QTL analysis. Genes were investigated to generate novel drug targets.

**Results:**

We identified 69 asthma SNPs that were associated with at least one trait, with 20 SNPs being associated with multiple traits. The SNP annotated to SMAD3 was the most pleiotropic. In total, 42 SNPs were associated with eosinophil counts, 18 SNPs with airway obstruction, and 21 SNPs with atopy. We identified genetically driven pathways regulating eosinophilia. The largest network of eosinophilia contained two genes (IL4R, TSLP) targeted by drugs currently available for eosinophilic asthma. Several novel targets were identified such as IL‐18, CCR4, and calcineurin.

**Conclusion:**

Many asthma SNPs are associated with blood eosinophil counts and genetically driven molecular pathways of asthma‐associated traits were identified.

## 1 INTRODUCTION

Asthma is a complex, heterogeneous inflammatory disease caused by a combination of genetic and environmental factors. It is characterised by respiratory symptoms, such as wheezing and chest tightness, and pathophysiological mechanisms, like reversible airway obstruction, and airway hyper‐responsiveness. Previously, asthma was regarded as a single disease entity. However, currently, distinct phenotypes of asthma are recognised due to an increased understanding of its underlying mechanisms. These asthma phenotypes are based on clinical presentation, inflammatory patterns, severity and triggers and/or pathophysiological mechanisms. Thus, studies into the pathogenic mechanisms of asthma should address this disease heterogeneity.^1^

Genome‐wide association study (GWAS) is an approach that can be used to reveal the underlying mechanisms by identifying single nucleotide polymorphisms (SNPs) that are associated with asthma. Recently, we described a list of 128 independent single nucleotide polymorphisms that were previously associated with asthma in European/white populations at a genome‐wide significant level.^2^ These SNPs are associated with asthma in general; however, it is unknown if these SNPs are involved in specific traits such as eosinophil counts, atopy or airway obstruction. Associations of asthma SNPs with a specific asthma‐associated trait may reveal a specific endotype, driven by a distinct molecular network.

It has been previously observed that many asthma SNPs associate with different asthma related phenotypes, indicating potential pleiotropic effects.^3^, ^4^ Analysing the association between asthma SNPs and asthma‐associated traits in the general population may therefore reveal insights into the genetic architecture of this disease. Alternatively, asthma SNPs may only act in a disease‐specific manner, which would result in associations with traits only in asthma populations, but not in the general population.

In this paper, we analysed the association of asthma SNPs with various associated traits in the general population as well as in asthma patients only; next, we performed expression quantitative trait loci (e‐QTL) and pathway analyses to reveal the downstream functional effects of SNPs associated with specific traits and test the hypothesis that these form distinct molecular pathways. Finally, we verify if the implicated genes were already targeted by known asthma drugs and propose new drug targets and drugs for asthma, by repositioning drugs for other indications based on genetic evidence.

## 2 MATERIAL AND METHODS

### 2.1 Study design

SNP selection was based on our previous review, where we performed a systematic analysis of GWAS to identify SNPs associated with asthma at genome‐wide significant level.^2^ This resulted in a list of 128 independent asthma‐associated SNPs, which we call asthma SNPs in the remainder of this paper.

These asthma SNPs were tested for association with seven asthma‐associated traits (ATs); blood eosinophil counts, forced expiratory volume 1st second (FEV_1_), (FEV_1_/forced vital capacity [FVC]), total Immunoglobulin E (IgE), skin prick test positivity (skin test to different inhalant allergens), severity of airway hyperresponsiveness (AHR), and childhood (<18 years) onset asthma using linear and logistic regression modelling, as appropriate.

For this study, we conducted the analyses in three independent Dutch general population and patient cohorts as shown in Table 1. The first is the Lifelines cohort (*n* = 32,817) which represents a general population cohort, comprising two and three‐generation families.^5^ In this cohort, we tested the association between asthma SNPs and eosinophil counts and airway obstruction (FEV_1_, FEV_1_/FVC) in both the general population and asthma patients. In addition, we tested the association between asthma SNPs and childhood‐onset asthma.^6^ The second cohort is the Vlagtwedde‐Vlaardingen (Vlw‐Vld) general population‐based cohort *n* = 1554.^7^ Here, we investigated the association of asthma SNPs with eosinophil counts, airway obstruction (FEV_1_, FEV_1_/inspiratory vital capacity [IVC]), atopy (total serum IgE, skin test) and AHR. Since no reliable asthma diagnosis was present in the Vlagtwedde‐Vlaardingen study, this cohort was not investigated in the asthma only analysis. The third cohort is the Dutch Asthma GWAS (DAG) cohort which consists of 917 asthma patients.^8^ In this cohort, we conducted the genetic association with eosinophil counts, airway obstruction (FEV_1_, FEV_1_/FVC), atopy (total serum IgE, skin test), AHR and childhood‐onset asthma. The three studies were approved by the Medical Ethics Committee of the University Medical Center Groningen and a written informed consent was provided by all participants. Detailed descriptions of these populations, the genetic datasets and phenotypes are provided in the Online Supplement S1. Meta‐analyses were subsequently conducted for blood eosinophil counts, FEV_1_, FEV_1_/VC and childhood‐onset asthma for cohorts representing the general population (Lifelines/Vlw‐Vld) and cohorts with asthma patients only (Lifelines, asthma patients only and DAG). Although the LifeLines study contributed the largest sample size to our study, other smaller cohorts provided a broader phenotypic assessment, such as BHR, skin tests and total IgE levels. We subsequently investigated the functional consequences of associated SNPs on gene expression by performing cis‐eQTL analysis using eQTLGen^9^ and GTEx.^10^ For each trait, the associated SNPs were annotated to eGenes by eQTL‐analysis, and we then performed functional enrichment, pathway and protein‐interaction analyses.

**TABLE 1:** Description of the Study population.

Characteristics	Lifelines(general)	*N*	Lifelines(asthma)	*N*	VLW‐VLD(general)	*N*	DAG (asthma)	*N*
Population	General	32,817	Asthma patients	2923	General	1554	Asthma patients	917
Age (*y*). Mean (min‐max)	42.9 (18–90)	32,817	40 (18–80)	2923	52.9 (35–80)	1554	34 (5–75)	917
Gender. Male (%)	40.8	32,817	40	2923	46.7	1554	46.9	917
Blood eosinophils log (ul/cell). Mean (SD)	2.17 (0.27)	32,252	2.25 (0.28)	2861	2.17 (0.27)	1515	2.18 (0.43)	769
FEV_1_(L). Mean (SD)	96 (12.6)	22,785	91 (13.7)	2042	86.6 (14.39)	1440	86.3 (20.7)	898
FEV_1_/FVC. Mean (SD)	0.77 (0.07)	22,789	0.75 (0.09)	2043	0.73 (0.09)	1440	0.79 (0.1)	263
Total IgE log (kU/L). Mean (SD)	n.a.		n.a.		1.43 (0.59)	1465	2.11 (0.66)	772
Skin prick test positivity (%)	n.a.		n.a.		219 (15.3)	1428	577 (85.2)	677
AHR slope log (SD)	n.a		n.a.		0.7 (0.73)	435	3.17 (1.44)	728
Childhood onset asthma^a^ *N* (%)	599 (75.9)	789	n.a.		n.a.		578 (80.4)	719

Abbreviations: N, number of subjects data field available for; n.a, not available; SD, standard of deviation.

^a^ Childhood onset asthma defined as a diagnosis occurring before the age of 18.

### 2.2 Statistical methods

Linear or logistic regression analyses of SNPs with asthma‐associated traits were performed in SAIGEgds,^11^ which accounts for the correlation between family members in the Lifelines cohort and Plink v1.90p^12^ (Vlw‐Vld and DAG) using an additive model. For the general population cohorts, age, sex and asthma were included as covariates. For the asthma cohorts, age (excluding the analysis of age of onset) and sex were included as covariates. Meta‐analyses were performed in Plink using a fixed model. We predefined two significance thresholds: adjusted Pvalue<0.05 (Bonferroni correction over 128 SNPs), and nominal, non‐adjusted *p* value < 0.05. The expected direction of the association was taken into consideration: positive association between the asthma‐associated risk allele and eosinophil counts, IgE, skin test, AHR, and childhood‐onset asthma and negative association between the asthma‐associated risk allele and FEV_1_, FEV_1_/FVC.

The functional consequences of the risk SNPs on gene expression were investigated using published cis‐eQTL datasets (eQTLGen, GTEx) from whole blood for SNPs associated with eosinophils, IgE, and childhood‐onset asthma, whole blood and lung for SNPs associated with airway obstruction and AHR, and whole blood and skin for SNPs associated with skin test. The selection of eGenes was based on the significance of *p*‐value below 2.3*10^−9^, as reported previously.^2^

Functional enrichment analysis was performed on eGenes associated with blood eosinophil counts, by mapping genes to g:GOST on g:Profiler (biit.cs.ut.ee/gprofiler) to detect statistically significant enriched terms. The data sources that were used in g:profiler are Gene Ontology (molecular function, biological process), KEGG, Reactome, Wikipathways, and Human Protein Atlas. For multiple testing correction, we used Benjamini Hochberg FDR below 0.05 as a threshold. For the term size, it was set to 200 since large pathways are of limited interpretative value. Next, eosinophil counts gene list was submitted to STRING (string‐db.org), to investigate significant gene co‐expression clustering and protein‐protein interaction. The minimum required interaction score was set to 0.7 (high confidence). Pathways and eGenes associated with eosinophil counts were submitted to open targets platform (platform.opentargets.org), to acquire information about potential targets, their drugs, and diseases.

## 3 RESULTS

### 3.1 Study populations

For the general population analyses (*N* = 25,371) we used Lifelines and VLW‐VLD cohorts and for the asthma population analyses (*N* = 3840) we used Lifelines (only asthma) and DAG cohorts.

The description of the study populations is shown in Table 1.

### 3.2 Genetic association with asthma‐associated traits

Out of the 128 asthma SNPs, a total of 123, 120, and 122 SNPs in LifeLines, Vlw‐Vld and DAG cohorts, respectively, were available for analysis.

In the general population, 38 asthma SNPs were associated with eosinophil counts, 16 of which had a *p*_adj_ < 0.05. In the asthma population, 14 SNPs showed nominal association with eosinophil counts, with 10 SNPs overlapping with the general population (rs449454 [*NDFIP1*], rs1342326 [*IL33*], rs3771180 [*IL1RL1*], rs7130588 [*LRRC32*], rs56375023 [*SMAD3*], rs13412757 [*LINC00299*], rs479844 [*ETS1*], rs3024655 [*IL4R*], rs16903574 [*FAM1*05A], rs11088309 [*RUNX1*]) and 4 SNPs were associated in the asthma population only (rs4233366 [*ADAMTS4*], rs11742240 [*IL7R*], rs1684466 [*NRROS*], rs2766667 [*ZNF217*]). In contrast to our expectation, 6 asthma SNPs showed nominal association with lower eosinophil counts in the general population (rs1233578 [*ZBED9*], rs519973 [*BCL6*], rs7686660 [*USP38*], rs6691738 [*TNFSF4*], rs2507978 [*HLA‐B*], rs3117098 [*HCG23*]), and 1 SNP in the asthma population (rs2786098 [*CRB1*]).

In the general population, 11 SNPs were associated with airway obstruction (FEV_1_ and/or FEV_1_/FVC), with 1 SNP overlapping between FEV_1_ and FEV_1_/FVC with a *p*_adj_ < 0.05 in FEV_1_. In the asthma population, 8 SNPs were nominally associated with airway obstruction, with 1 SNP that overlapped.

**FIGURE 1:**
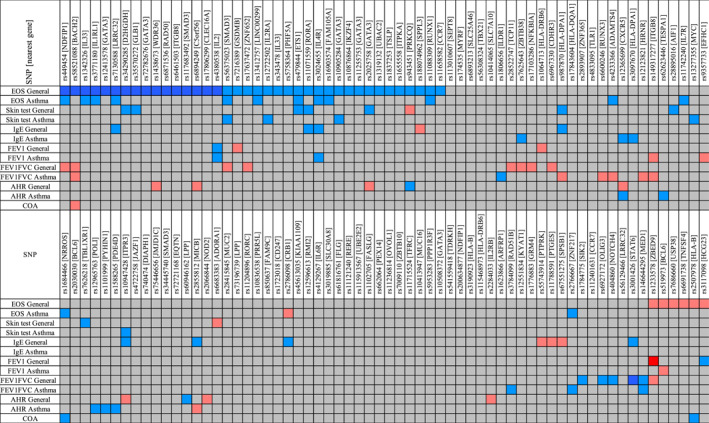
Association of asthma single nucleotide polymorphisms (SNPs) with asthma‐associated traits. The blue and red colours display the direction of the BETA value. Asthma‐associated allele is taken as the risk allele: blue shows higher levels (positive association) and red shows lower levels (negative association). The colour intensity represents the significance of the association between the SNP and the phenotype (Dark red or dark blue: *p* adj < 0.05; Light red or light blue: *p* < 0.05).

For atopy, AHR, and childhood‐onset, a lower number of asthma SNPs showed association. Atopy is represented by skin test and total serum IgE. In total, there were 21 SNPs nominally associated with atopy with 3 SNPs overlapping between skin test and total IgE. For skin test, 8 SNPs were nominally associated in the general population, and 5 SNPs in the asthma population. For total IgE, 8 SNPs were nominally associated in the general population and 3 SNPs in the asthma population. For AHR, two SNPs were nominally associated in the general population, and 5 SNPs in the asthma population. Finally, two SNPs were nominally associated with childhood‐onset asthma, and 1 SNP showed a negative nominal association with this sub‐type. A visual representation of these results is provided in Figure 1, whereas full results can be found in the Online Supplementary Tables S1 and S2.

#### 3.2.1 Pleiotropy

**FIGURE 2:**
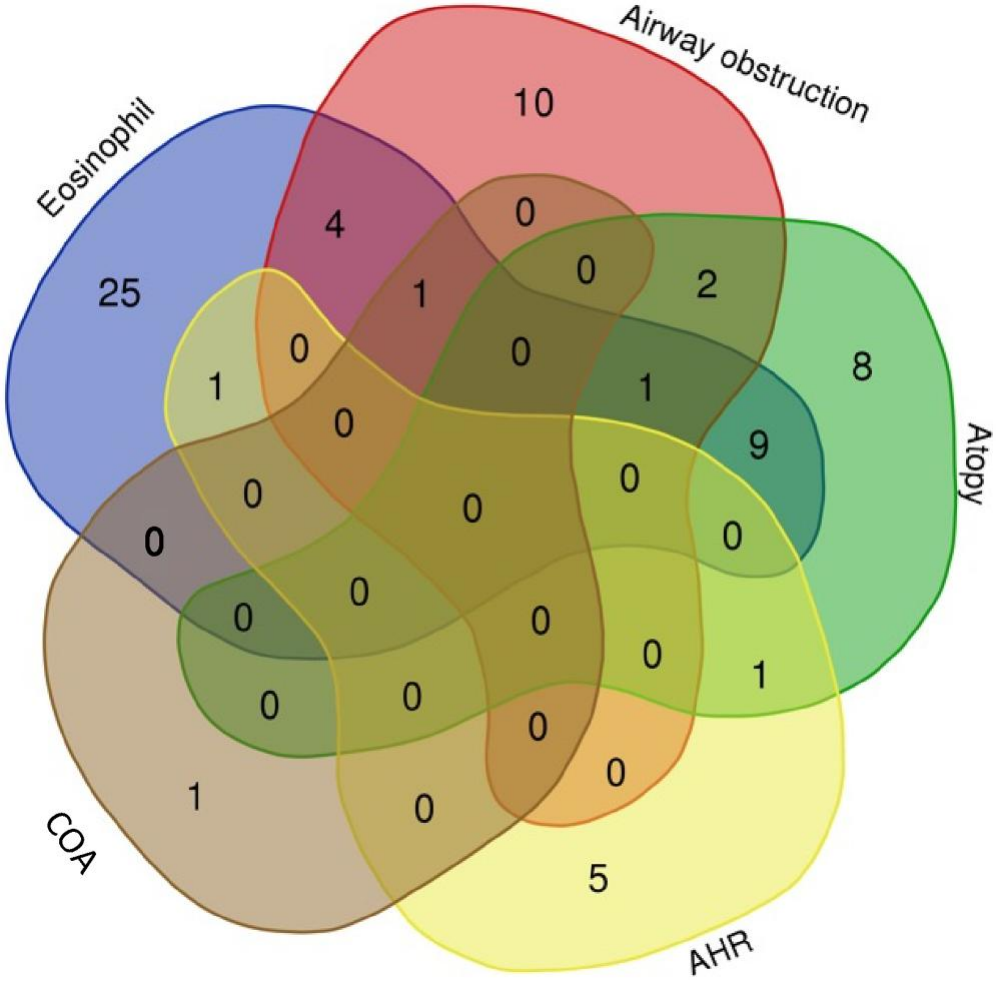
Overlap of single nucleotide polymorphisms (SNPs) associated with asthma‐associated traits. AHR, Airway hyper‐responsiveness; COA, Childhood‐onset asthma.

Whereas all asthma SNPs were selected based on prior evidence of their association with asthma, we also inspected further overlap in association with associated traits (Figure 2, Supplementary Table S3). We observed that there is 1 SNP (rs56375023, located in *SMAD3*) that was related to three associated traits: blood eosinophil counts, airway obstruction and atopy. We observed that 9 asthma SNPs were associated with eosinophil counts and atopy (located in or near *LRRC32, D2HGDH, GLB1, IL2RA, ETS1, RORA, IL4R, GATA3* genes) and five SNPs with eosinophil counts and airway obstruction (located in or near *NDFIP1, BACH2, GSDMB, ZNF652, ADAMTS4* genes).

#### 3.2.2 Disease specificity

**FIGURE 3:**
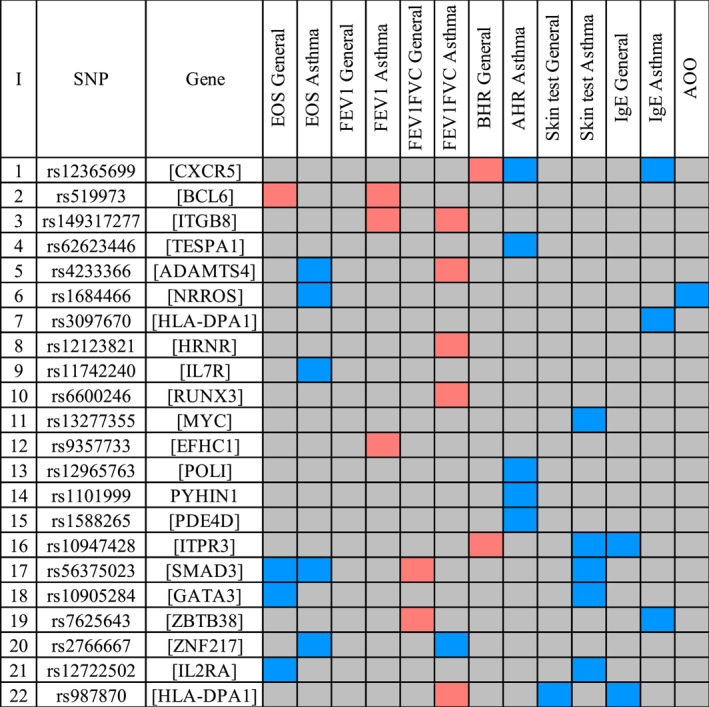
Asthma‐specific single nucleotide polymorphisms (SNPs). The blue and red colours display the direction of the BETA value. Asthma‐associated allele is taken as the risk allele: blue shows higher levels (positive association) and red shows lower levels (negative association). The colour intensity represents the significance of the association between the SNP and the phenotype (Dark red or dark blue: *p* adj < 0.05; Light red or light blue: *p* < 0.05).

We observed several asthma SNPs to be associated with one or more asthma‐associated traits in asthma patients, but not in the general population (Figure 3). These SNPs are located in or near *BCL6, ITGB8, TESPA1, ADAMTS4, NRROS, HLA‐DPA1, HRNR, IL7R, RUNX3, MYC, EFHC1, POLI, PYHIN1, PDE4D, ITPR3, SMAD3, GATA3, ZBTB38, ZNF217, and IL2RA*. Of interest, one asthma‐specific SNP (rs12365699), near *CXCR5* gene, showed a different direction of effect in the asthma population (risk allele was associated with higher AHR), whereas in the general population, this SNP was associated with lower AHR.

#### 3.2.3 Functional genetics

After conducting the cis‐eQTL analysis, gene expression levels of 120 unique genes were significantly associated with SNPs that showed association with asthma traits. For SNPs associated with blood eosinophil counts, we identified 82 eGenes. Amongst these genes, we identified genes encoding drug targets for eosinophilic asthma, such as *IL4RA* and *TSLP*. Some potential targets and their role in eosinophilia are mentioned in Supplementary Table S4.

We identified 7 eGenes (*MICA, HLA‐DRB5, HLA‐DQA2, HLA‐DRB6, INPP4B, USP38, TNFSF4*) of SNPs associated with lower blood eosinophil counts. SNPs associated with airway obstruction, atopy, AHR and age of onset had 38, 48, 3 and 1 eGenes, respectively (Supplementary Table S5).

#### 3.2.4 Network and pathway analysis

**FIGURE 4:**
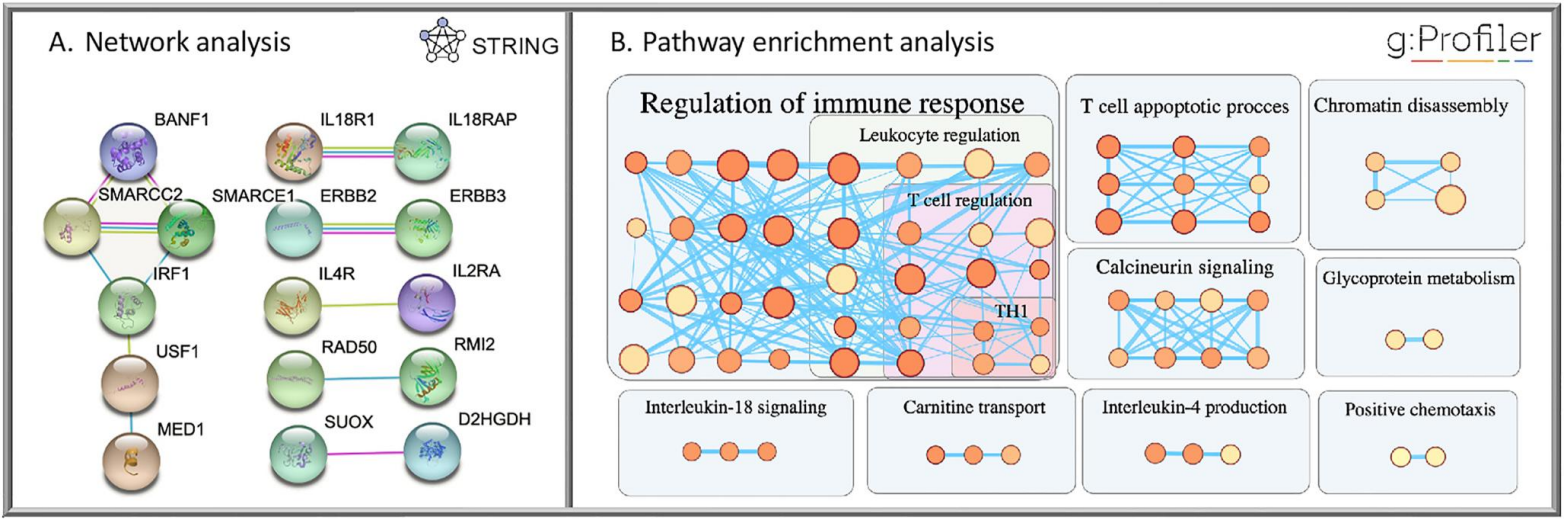
Eosinophil counts pathway and network analysis. (A) Physical interaction subnetwork of genes associated with eosinophilia: The Nodes represent proteins while the edges represent protein‐protein interaction. (B) High eosinophil counts enrichment pathway terms: Pathway terms (circles) are clustered based on words in common and genes overlapped. The circle colour and size reflect the enrichment score and the term size respectively. The edges represent the genes shared between the terms. TH1, T‐helper1 immune response.

In the functional enrichment analysis, 114 term names were generated for blood eosinophil counts via g:Profiler (Supplementary Table S6). The most relatively specific repeated terms in the enrichment analysis were: T cells (differentiation, activation, and apoptotic process), T‐helper Type 1 response, Interleukin‐4, Interleukin‐18 pathways, and Calcineurin signalling as shown in Figure 4.

## 4 DISCUSSION

Asthma is a complex, polygenic, heterogeneous inflammatory disease. One hypothesis is that this polygenic origin may relate to the broad disease definition used in genetic studies; with the outcome ‘asthma’ capturing multiple disease endotypes. Our analysis shows that many asthma SNPs have associations with asthma‐associated traits. In our study, we show a clear association of asthma SNPs with eosinophil counts and, to a lesser degree, with airway obstruction. We report fewer associations of asthma SNPs with total IgE, skin test and AHR. Unfortunately, fractional exhaled nitric oxide (FeNO), which is another interesting type 2 inflammatory biomarker, was not present in the cohorts under study. We observed pleiotropic effects of 20 asthma SNPs but also observed that 22 SNPs had an association with an associated trait in asthma patients only. Our results, therefore, provide evidence for pleiotropic, as well as trait‐specific effects. Based on the shared association of asthma SNPs with blood eosinophil counts, we identified genetically driven pathways that may regulate blood eosinophil counts in asthma, which could be targets of future interventions for eosinophilic asthma.

Blood eosinophil counts were associated with 38 asthma SNPs in the general population. Our findings are consistent with previous reports in studies on subjects of European ancestry, in which nearly 84% of the asthma SNPs showed association with eosinophil counts^13^, ^14^ (Supplemental Table S7). Ten asthma SNPs that were associated with blood eosinophil counts in the general population were also associated with eosinophil counts in asthma. These asthma‐associated SNPs may constitute genetic susceptibility to eosinophilic asthma; as blood eosinophilia correlates with airway eosinophilia in asthma patients.^15^ However, we recognise that we might have underestimated the number of these SNPs, as we take statistical significance as the main criterium of association and were relatively underpowered in the asthma cohorts compared to the general population. Therefore, we present the nominal *p*‐value together with direction of effect in the Supplementary Tables S1 and S2 to better assess the consistency of the results.

Although all 128 SNPs were previously reported to be associated with asthma at genome wide significance in other populations, we observed that some SNPs were not associated with asthma related traits in our included cohorts. This may be due to lack of power, or that specific related phenotypes for that SNP (i.e., neutrophilic asthma) were not investigated in our study. Alternatively, association with asthma related traits may be partly due to residual confounding, although the statistical model in the general population accounted for the presence of asthma. Moreover, since the publication of our review, additional asthma SNPs have been proposed; (Olafsdottir et al. 2020, Han et al. 2020); we propose that future approaches should include these genes.

We observed SNP associations with asthma‐associated traits in the asthma population only, but not in the general population. These genes annotated from the SNPs are referred to as asthma‐specific targets. In blood eosinophil counts, there were 8 genes (*FCER1G, IL7R, USF1, APOA2, NDUFS2, ZNF217, PPOX, TSHZ2, TOMM40L*) annotated from 4 SNPs that associate with the trait only in the asthma population. Some of these genes are known for their role in eosinophilic inflammation. FCER1G is part of the high‐affinity IgE tetramer receptor Fc Epsilon Receptor I (FcεRI). IgE‐activated mast cells are the main producers of prostaglandin D2 (PGD2), which stimulate eosinophils.^16^ Another asthma‐specific target is IL‐7R, which acts as a receptor for interleukin‐7 (IL‐7) and TSLP. IL‐7 and TSLP may contribute to airway inflammation by promoting activation and survival of eosinophils.^17^ We also observed potential targets such as PPOX, TOMM40L and NDUSF1 that are involved in mitochondrial dysfunction and oxidative stress which when activated in the inflammatory cells lead to tissue injury in the bronchial epithelium.^18^

One asthma SNP had strongly pleiotropic effects. This SNP rs56375023 *[SMAD3]* was associated with blood eosinophil counts in both the general and asthma populations, low FEV_1_/FVC in the general population and IgE sensitisation in the asthma population. SMAD3 is an intracellular transducer that mediates TGF‐ß signalling. TGF‐ß is a multifunctional cytokine. It has anti‐inflammatory roles inhibiting Th1 and Th2 differentiation and maturation of macrophages and dendritic cells, and it also induces T regulatory cells.^19^, ^20^, ^21^, ^22^ On the other hand, TGF‐ß has a proinflammatory effect by enhancing leucocyte recruitment, IgA production, chemoattracting leucocytes and modulating adhesion molecules.^23^, ^24^ A study on *SMAD3* knockout mice revealed increases in Th2 and IL17 proinflammatory pathways and also induced regulatory elements like Foxp3.^25^ TGF‐ß also plays a role in airway remodelling and structural cell proliferation via Smad3 which leads to airway obstruction and AHR.^26^ Our results on pleiotropic genes may explain the correlation between these different traits in asthma, either by genes playing a pleiotropic role in the development of these phenotypes or through causal pathways, which would imply that one trait causes the occurrence of another trait.

We generated asthma trait‐associated lists of SNPs that were annotated to potential drug targets. It has been previously shown that the success of drug development doubles when drug targets have genetic evidence, compared to those without genetic evidence.^27^ Two eGenes related to SNPs for eosinophilic asthma encode for drug targets for eosinophilic, type 2 asthma: *IL4R* (dupilimab) and *TSLP* (tezepelumab).^28^ These findings support the validity of our genomics‐guided drug discovery approach and suggest that some of the other genes identified may represent novel drug targets for eosinophilic asthma (Table 2), such as *IL18, CCR4* and calcineurin.

**TABLE 2:** Genes associated with eosinophil counts and their drugs.

Gene	Eosinophil	Atopy	AWO	Drugs	Target	Disease
*IL4R*	General/Asthma	General	Asthma	Dupilumab	*IL4R*	Asthma
				Pascolizumab	*IL4*	Asthma
*IL7R*	Asthma			Tezepelumab,AMG‐157	*TSLP*	Asthma
*IL1RL1*	General/Asthma			Astegolimab	*IL1RL1*	Asthma
				Etokimab, Itepekimab	*IL33*	Asthma
*FCER1G*	Asthma		Asthma	Omalizumab, Ligelizumab, Quilizumab	*IGHE*	Asthma
*NDFIP1*	General/Asthma		General	Mepolizumab, Reslizumab	*IL5*	Asthma
*CCR7*	General			Navarixin	*CXCR1,CXCR2, CCR7*	Asthma
*CCR4*	General	General		Mogamulizumab	*CCR4*	Asthma (Terminated)
*IL2RA*	General	Asthma		Daclizumab	*IL2RA*	Asthma
				Basiliximab	*IL2RA*	Diabetes mellitus
*IL18RAP*	General/Asthma			GSK1070806, Medi‐2338	*IL18*	Diabetes mellitus, COPD
*IL18R1*	General/Asthma					
*NDUFS2*	Asthma		Asthma	Metformin	*NDUFS2*	Diabetes mellitus
*MAP3K11*	General/Asthma	General		CEP‐1347	*MAP3K11*	Parkinson disease
				Dactolisib	*PIK3R1*	Respiratory tract infections
*RPS26*	General			Ataluren	*RPS26*	Cystic fibrosis
*GATA3*	General	Asthma		Pyrrothiogatain	*GATA3*	Leukaemia
*PRKCQ*	General			Midostaurin	*PRKCQ*	Leukaemia
*CDK12*	General		General	At‐7519	*CDK12*	Leukaemia
*PSMD3*	General		General	Oprozomib	*PSMD3*	Multiple myeloma
*ERBB2*	General		General	Pertuzumab	*ERBB2*	Breast cancer
*PDCD1*	General		General	Pembrolizumab	*PDCD1*	Squamous cell carcinoma

Abbreviation: AWO, airway obstruction.

IL‐18 receptors (IL18R1 and IL18RAP) share the same loci 2q12 with IL33 receptor (IL1RL1) but not given much consideration. Previous studies show IL‐18 critical role in eosinophil development and transformation from naïve to pathogenic eosinophils in patients with allergic diseases.^29^ IL‐18 pathway can be blocked by targeting IL‐18 with two drugs (GSK1070806, MEDI‐2338) in clinical trials positioned for Diabetes mellitus and COPD respectively. CCR4 receptors are expressed on Th2 lymphocytes that secrete eosinophil‐related cytokines such as IL‐5, IL‐4, and IL‐13.^30^ CCR4 is an interesting target blocked by the drug Mogamulizumab which was launched in 2012 for CCR4‐positive adult T‐cell leukaemia‐lymphoma. In 2014, the drug was repositioned for asthma but discontinued.^31^ Finally, calcineurin signalling regulates eosinophil progenitor production and therefore contributes to the development of eosinophilia in allergic asthma.^32^ Cyclosporin and tacrolimus are calcineurin inhibitors used for solid organ transplantation. Cyclosporin has been positioned for steroid‐dependent asthma and showed improvement of lung function.^33^ Next to cyclosporin, tacrolimus was used to treat severe asthma; however, there is not enough data to confirm efficacy.^33^ Moreover, the application of calcineurin inhibitors in asthma is limited because of their systemic adverse effects, which may be limited if these drugs could be applied locally.

Currently, there is an unmet need for drugs positioned for non‐eosinophilic asthma. In this study, we have identified SNPs associated with low eosinophil counts. One of the interesting genes is *TNFSF4* (OX40L) which plays a role in modulating airway inflammation in asthma. Oxelumab is an anti‐OX40L, which was tested in a small clinical trial for adults with mild allergic asthma without clear beneficial effects.^34^ However, our results suggest to test this drug in non‐eosinophilic, type 2 low asthma.

In conclusion, many asthma‐associated GWAS SNPs have distinct associations with asthma traits, such as eosinophilia, airway obstruction and atopy. We found evidence for pleiotropic genes, but also some disease‐specific genes were identified. This study shows that there are distinct sets of asthma SNPs that constitute distinct molecular pathways, according to protein interaction networks analysis. We identified genetically driven pathways regulating eosinophilia in asthma, with two genes (*IL4R, TSLP*) targeted by drugs currently available for eosinophilic asthma.

## AUTHOR CONTRIBUTIONS

**Zaid W. El‐Husseini:** Conceptualization (supporting); Formal analysis (lead); Investigation (lead); Methodology (equal); Software (equal) Validation (equal); Visualization (equal); Writing – original draft (lead); Writing – review & editing (equal). **Judith M Vonk:** Data curation (supporting); Formal analysis (supporting); Supervision (supporting); Visualization (supporting). **Maarten van den Berge:** Data curation (lead); Resources (lead); Visualization (supporting). **Reinoud Gosens:** Formal analysis (Supporting); Investigation (supporting); Methodology (supporting); Project administration (supporting); Supervision (supporting); Visualization (equal); Writing – review & editing (supporting). **Gerard H. Koppelman:** Conceptualization (lead); Data curation (equal); Investigation (equal); Methodology (lead); Project administration (lead); Resources (supporting); Supervision (lead); Visualization (lead); Writing – original draft (supporting); Writing – review & editing (lead).

## CONFLICT OF INTEREST STATEMENT

Mr. El‐Husseini has nothing to disclose. Dr. Gosens reports grants from Marie Sklodowska‐Curie, during the conduct of the study; grants from Aquilo, grants from Boehringer Ingelheim, grants from Sanofi‐Genzyme, outside the submitted work. Dr. Vonk has nothing to disclose. Dr. Van den Berge reports grants from Chiesi, grants from GlaxoSmithKline, grants from Novartis, grants from Genentech, grants from Roche, outside of the submitted work. Dr. Koppelman reports grants from Marie Sklodowska‐Curie during the conduct of the study; grants from TEVA the Netherlands, GSK, Lung Foundation of the Netherlands, VERTEX, UBBO EMMIUS foundation, TETRI Foundation, and ZON‐MW outside the submitted work; and GHK participated in advisory board meetings for GSK, Astra Zeneca and PURE‐IMS (money to institution).

## Supporting information

Supporting Information S1

Supporting Information S2

## Data Availability

The data that support the findings of this study are available from Lifelines. Restrictions apply to the availability of these data, which were used under license for this study. Data are available from the authors with the permission of Lifelines.
