# Supplementary material for: Association of asthma genetic variants with asthma‐associated traits reveals molecular pathways of eosinophilic asthma
Source: Clin Transl Allergy. 2023 Apr 20;13(4):e12239. doi: 10.1002/clt2.12239 (PMC10119226; doi:10.1002/clt2.12239)
Supplement: Supplementary file 1 — Supporting Information S1 [file CLT2-13-e12239-s001.docx]

**Supplemental material**

***Association of asthma genetic variants with asthma-associated traits reveals molecular pathways of eosinophilic asthma***

**Methods**

**Lifelines[1]**

Lifelines is a multi-disciplinary prospective population-based cohort study examining in a unique three-generation design the health and health-related behaviours of 167,729 persons living in the North of The Netherlands. It employs a broad range of investigative procedures to assess the impact of biomedical, socio-demographic, behavioural, physical, and psychological factors on multi-morbidity, and complex genetics. The study was approved by the Medical Ethics Committee of the University Medical Center Groningen and a written informed consent was provided by all participants.

*Phenotype*

Asthma was defined as having doctor’s diagnosis of asthma or taking asthma medication while having 2 or more of the following asthma symptoms: wheeze, attacks at rest, woken by an attack.

Eosinophil count were measured using Automated Hematology Blood Analyzer (XE2100–system; Sysmex, Japan). In the main analyses, absolute eosinophil count was utilized. Absolute eosinophil count was calculated by multiplying the relative eosinophil count by the total leukocyte count.

FEV_1_ and FVC were measured by a spirometer following the ERS/ATS guidelines [2]. According to the test criteria the difference between the best and the next best FEV_1_ and FVC should not exceed 150 ml. If the difference was greater than 150 ml, the test was repeated.

Age of onset (AOO) was determined by participants answers to the question “How old were you when you had your first asthma attack?”.

*Genotyping*

A subset of 38,030 Participants were genotyped on the Infinium Global Screening Array® (GSA) MultiEthnic Disease Version. Subjects were selected based on richness of phenotypic information, in combination with informativeness within the family based design, as described in [3]. A standard quality control was performed on both samples and markers, SNPs were excluded with a low genotype rate (<99%), variants showing deviation from Hardy-Weinberg equilibrium p < 10^−6^ or excess of Mendelian errors in families >1% of the parent-offspring pairs. Samples that did not show consistent information between reported sex and genotypes on the X chromosome, between reported familial information and observed identity-by-descent sharing with family members, and between genotypes available from this and previous studies were excluded. In total of 36,339 samples and 571,420 SNPs were available. Imputation was done using the HRC panel version 1.1 as implemented by the Sanger Imputation Service at the time (24-09-2019).

*Statistical methods*

The selected 128 asthma SNPs were extracted from the genotyped data.

From the 128 asthma SNPs: 33 SNPs were genotyped, 76 were imputed, 14 had LD with the asthma SNPs (table1), and 5 were not included. SAIGEgds was used to perform association analysis between the asthma SNP and asthma-associated traits (eosinophil counts, FEV_1_, FEV_1_/FVC and childhood-onset asthma) in both the total Lifelines (general) population and the asthma population. SAIGEgds corrects for Family, cryptic relatedness, population stratification and case control imbalance. For the general population, sex, age, and asthma covariant sets were used, and for the asthma population, age and sex (except for childhood-onset asthma which was adjusted for sex only). Smoking status is not a confounder and thus was not adjusted for in the model. A sensitivity test was done to confirm that the exclusion of smoking status does not affect the p value of the SNPs association with FEV1 and FEV1/FVC in Life Lines cohort (figure S1).

For eosinophils, the unit 10E9/L was converted to cells/ul e.g. (0.17->170), 10 units were added to each sample since some of the values were 0, and the ln (natural logarithm) was taken to obtain a normal distribution. Linear regression modelling was used for ln (eosinophil counts), FEV_1_/FVC % predicted, and FEV_1_% predicted. Childhood-onset asthma was analysed as a binary trait with cases being defined as having the first asthma attack occurring before the age of 18y . The script can be found here: <https://github.com/molgenis/GWAS_UMCG_HPC/tree/main/SAIGEgds>

**Vlagtwedde and Vlaardingen Cohort[4]**

The Vlagtwedde-Vlaardingen started in 1965 as the first epidemiological cohort study in the Netherlands, and the final visit was in 1990. The study consists of over 8000 participants from Vlagtwedde and Vlaardingen, representing two different regions in the Netherlands. For the current analyses only data from the final visit were used since DNA is only available for subjects who completed this visit. The database contains DNA isolated from peripheral white blood cells, questionnaires and clinical data. The study protocol was approved by the Medical Ethics Committee of the University Medical Center Groningen and a written informed consent was provided by all participants.

*Phenotype definition*

In the Vlagtwedde–Vlaardingen study, asthma was defined a positive answer of the participant to the question “Have you ever had attacks of shortness of breath with wheezing at rest?” which was used as a co-variate in the model. In this cohort, blood eosinophil counts were assessed at baseline in a 1:11 dilution of peripheral blood with a Bürker counting chamber. inspiratory vital capacity (IVC) was used instead of FVC and measured using a water-sealed spirometer (Lode Spirograph D52, Lode, Groningen, the Netherlands). Spirometry measurements were performed according to the ATS/ERS criteria. Skin prick tests included six inhalant allergens: house dust mite, mixed grass pollen, mixed tree pollen, dog epithelium, cat epithelium, and mould (Aspergillus fumigatus). Skin test positivity was defined as one or more positive skin prick tests. Total serum IgE concentrations were determined with the CAP system (Pharmacia, Woerden, The Netherlands) and expressed in kU/L. Bronchial responsiveness to histamine was assessed by the method of Tiffeneau as modified by De Vries [5], which meets standardization guidelines. The severity of AHR is measured by a slope which is calculated by dividing the difference between FEV_1_ at baseline and the FEV_1_ at the final dose step at which the 20% fall of FEV_1_ was reached by the dose that was taken at that point.

*Genotyping*

In Vlagtwedde and Vlaardingen Cohort, blood samples were genotyped using Illumina CytoSNP-12 arrays. SNPS with low genotype rate (<95%), a minor allele frequency < 1%, and a Hardy-Weinberg P value less than 10^−4^ were excluded. Samples from first-degree relatives and non-white subjects were excluded based on self-reporting, outlier (identity-by-state), and principal components analysis (PCA).

*Statistical methods*

Six separate analyses were performed to assess the genetic association with eosinophil counts, FEV_1_/IVC % predicted, FEV_1_% predicted, total IgE, skin test and severity of AHR (AHR slope).

Eosinophils counts were converted to cells/ul and then the natural logarithm was taken. The AHR slope and total IgE were also ln-transformed.

The 128 asthma SNPs were extracted from the genotyped data. From the 128 asthma SNPs : 14 SNPs are genotyped, 97 imputed, 9 have high LD with the top SNPs, and 8 are not included.

PLINK 1.9 was used to preform association analysis between selected SNP and asthma Phenotypes: natural logarithm of eosinophil counts (cells/ul), FEV_1_/IVC % predicted, FEV_1_ % predicted, severity of AHR (natural logarithm of AHR slope), Natural logarithm of total IgE, and skin test. Age, sex, and asthma are used as covariant sets. Linear regression modelling was used for eosinophil counts, FEV_1_/IVC % predicted, FEV_1_ % predicted, severity of AHR, and total IgE. Logistic regression was used for skin test positivity.

**Dutch Asthma GWAS cohort (DAG)**[6]

The DAG cohort Genotype is a combination of two different studies: a trio study of 468 proband with asthma and two family members; with a case-control study of 452 asthmatics and 511 controls. For this analysis, we selected the asthma patients only. The study was approved by the Medical Ethics Committee of the University Medical Center Groningen and a written informed consent was provided by all participants

*Phenotype*

Asthma was defined as a combination of doctor’s diagnosis of asthma, asthma symptoms and AHR.

Eosinophils in peripheral blood were measured by a counting chamber and total IgE by ImmunoCAP™. Spirometry measurements were performed with a water-sealed spirometer. AHR was tested using tidal breathing with a stimulus histamine or methacholine. The severity of AHR is measured by a slope which is calculated by dividing the difference between FEV_1_ at baseline and the FEV_1_ at the final dose step at which the 20% fall of FEV_1_ was reached by the dose that was taken at that point. Age of onset was assessed from a self-reported questionnaire or medical history report. Atopy was measured by intracutaneous skin-tests for subjects older than 12 years or a skin prick test for children younger than 12 years. The DAG cohort is made of several studies, each study conducted various combinations of allergens.[6]

*Genotyping and imputation:*

The DAG cohort was genotyped using the Illumina 317 Chip and the Illumina 370 Duo Chip (Illumina, San Diego, CA). Quality control was performed per chip by excluding participants with missing genotype call rate (>0.01), non-Caucasian subjects and related individuals. SNPs were excluded with a missing genotype rate (>0.01), a MAF <0.01 and a Hardy-Weinberg equilibrium P-value <10^-7^. Afterwards the two chips were merged together and SNPs that were not available in both cohorts were excluded. In total there are 294,775 SNPs. The imputation was executed using IMPUTE 2 with EUR panel as the reference data of the 1000 Genome project.

*Statistical methods*

The asthma SNPs were extracted from the genotyped data. From the 128 asthma SNPs: 22 SNPs are genotyped, 91 are imputed, 9 have high LD with the asthma SNPs, and 6 are not included.

PLINK 1.9 was used to perform association analysis between the selected SNPs and the asthma-associated traits: Natural logarithm of eosinophil counts (cells/ul), FEV_1_/FVC % predicted, FEV_1_ % predicted, natural logarithm of AHR slope, natural logarithm of total IgE, childhood-onset asthma and skin test positivity). Age and sex are used as covariant sets, except for childhood-onset asthma which adjusted for sex only. Linear regression modelling was used for eosinophil counts, FEV_1_, FEV_1_/FVC, AHR, total IgE, and logistic regression for childhood-onset (with cases being defined as having asthma diagnosis occurring before the age of 18y) and skin prick test positivity.

*Meta-Analysis*

A meta-analysis was conducted for eosinophil count, FEV_1_, FEV_1_FVC and childhood-onset asthma for cohorts derived from the general population (Lifelines /Vlw-Vld) and for cohorts derived from asthma populations (Lifelines Asthmatics/DAG) in PLINK 1.9 using a fixed model. The fixed model assumes that all studies included in a meta-analysis are estimating a single true underlying effect.

***Table1: Asthma top-SNPs and the LD SNPs in the cohorts***

| Top SNP | Lifelines | R^2^ | Top SNP | DAG | R^2^ | Top SNP | VlwVla | R^2^ |
| --- | --- | --- | --- | --- | --- | --- | --- | --- |
| rs3097670 | rs3135020 | 0.99 | rs541559418 | rs185187161 | 1 | rs541559418 | rs150407922 | 1 |
| rs541559418 | rs150407922 | 1 | rs1101999 | rs856091 | 1 | rs2507978 | rs114333358 | 1 |
| rs1102705 | rs2422254 | 0.92 | rs1233578 | rs6456834 | 0.94 | rs28895016 | rs149130795 | 1 |
| rs7625643 | rs6763927 | 0.97 | rs2507978 | rs114333358 | 1 | rs987870 | rs114788682 | 0.95 |
| rs113010607 | rs803054 | 0.72 | rs28895016 | rs149130795 | 1 | rs3097670 | rs114305667 | 1 |
| rs28895016 | rs9348876 | 0.97 | rs3097670 | rs114305667 | 1 | rs149317277 | rs4473914 | 1 |
| rs67551275 | rs2310923 | 0.7 | rs34445740 | rs10152593 | 1 | rs34445740 | rs10152593 | 1 |
| rs149317277 | rs4473914 | 1 | rs12965763 | rs143417371 | 1 | rs12965763 | rs76506978 | 1 |
| rs188074962 | rs2649998 | 0.99 | rs5758364 | rs79008 | 1 | rs5758364 | rs5758343 | 0.977 |
| rs1319132 | rs912130 | 1 |  |  |  |  |  |  |
| rs34445740 | rs10152593 | 1 |  |  |  |  |  |  |
| rs12965763 | rs76506978 | 1 |  |  |  |  |  |  |
| rs5758364 | rs5758343 | 0.977 |  |  |  |  |  |  |
| rs5953283 | rs4824751 | 1 |  |  |  |  |  |  |

Figure S1: Sensitivity test for FEV1 and FEV1/FVC in Lifelines

## Supplementary references

[1] S. Scholtens *et al.*, “Cohort Profile: LifeLines, a three-generation cohort study and biobank,” *Int. J. Epidemiol.*, vol. 44, no. 4, pp. 1172–1180, Aug. 2015, doi: 10.1093/IJE/DYU229.

[2] K. F. Chung *et al.*, “International ERS/ATS guidelines on definition, evaluation and treatment of severe asthma,” *Eur. Respir. J.*, vol. 43, no. 2, pp. 343–373, Feb. 2014, doi: 10.1183/09031936.00202013.

[3] E. A. Lopera Maya *et al.*, “Lack of Association Between Genetic Variants at ACE2 and TMPRSS2 Genes Involved in SARS-CoV-2 Infection and Human Quantitative Phenotypes,” *Front. Genet.*, vol. 11, Jun. 2020, doi: 10.3389/FGENE.2020.00613.

[4] D. A. van der Plaat *et al.*, “Genome-wide association study on the FEV 1/FVC ratio in never-smokers identifies HHIP and FAM13A,” *J. Allergy Clin. Immunol.*, vol. 139, no. 2, pp. 533–540, Feb. 2017, doi: 10.1016/J.JACI.2016.06.062.

[5] de VRIES, J. T. GOEI, H. BOOY-NOORD, and N. G. ORIE, “Changes during 24 hours in the lung function and histamine hyperreactivity of the bronchial tree in asthmatic and bronchitic patients,” *Int. Arch. Allergy Appl. Immunol.*, vol. 20, pp. 93–101, 1962, doi: 10.1159/000229248.

[6] M. A. Nieuwenhuis *et al.*, “Combining genomewide association study and lung eQTL analysis provides evidence for novel genes associated with asthma,” *Allergy Eur. J. Allergy Clin. Immunol.*, vol. 71, no. 12, pp. 1712–1720, Dec. 2016, doi: 10.1111/all.12990.
